# Editorial: Advances in the prevention and treatment of sudden cardiac death

**DOI:** 10.3389/fmed.2023.1335398

**Published:** 2023-12-01

**Authors:** Sebastian Schnaubelt, Enrico Baldi, Patrick Sulzgruber

**Affiliations:** ^1^Department of Emergency Medicine, Medical University of Vienna, Vienna, Austria; ^2^PULS – Austrian Cardiac Arrest Awareness Association, Vienna, Austria; ^3^Division of Cardiology, Fondazione IRCCS Policlinico San Matteo, Pavia, Italy; ^4^Cardiac Arrest and Resuscitation Science Research Team (RESTART), Fondazione IRCCS Policlinico San Matteo, Pavia, Italy; ^5^Division of Cardiology, Department of Internal Medicine II, Medical University of Vienna, Vienna, Austria

**Keywords:** sudden cardiac arrest, sudden cardiac death, emergency medicine, critical care, cardiopulmonary resuscitation, extracorporeal membrane oxygenation (ECMO)

As highlighted by a recent Lancet Commission, sudden cardiac death (SCD) is a continuous challenge for healthcare systems worldwide (1). The exact nomenclature varies, and sometimes SCD and out-of-hospital cardiac arrest (OHCA) are used synonymously, with the latter being a broad umbrella term. Be that as it may, the incidence of OHCA alone in Europe currently lies between 67 and 170 per 100,000 people, with cardiopulmonary resuscitation (CPR) attempted in about 50–60%, and survival rates (to hospital discharge) of only between 0 and 18%. Simultaneously, crucial bystander CPR is still not performed in the majority of cases, and the percentage of automated external defibrillator (AED) use is even worse (2). The often scarce resources sometimes appear not to be allocated optimally, and various resources settings must be included in the bigger picture (3). Apart from a few examples, even many high-income countries apparently choose to ignore the magnitude of the problem, and still rely heavily on the work of non-governmental organizations to at least cover the basics like layperson CPR education and awareness campaigning (4, 5). This is surprising as the burden of SCD is high in every aspect, not only to patients and relatives, but also economically. Despite this, and in spite of the chronic underfunding of cardiac arrest research, fortunately there is a motivated scientific community tackling the mentioned challenges. SCD research includes a wide spectrum of topics, ranging from animal studies all the way to randomized controlled trials of novel treatment options in humans. The Research Topic at hand was developed to promote the visibility of this spectrum.

## Advances in ventilation during CPR

Xu et al. presented a swine CPR model and evaluated a combination of chest compression synchronized ventilation (CCSV) and aortic balloon occlusion (ABO) as a novel approach to improve outcomes. They found that PaO_2_ was significantly higher in the CCSV groups (CCSV and CCSV + ABO) than in the non-CCSV controls, and improved myocardial and neurological function were seen in the CCSV + ABO group in the post-CPR phase. This is a further positive development after initial positive reports on CCSV (6), and the topic calls for a large randomized trial in humans to further evaluate its effects—maybe also in combination with other hemodynamic support.

## Extracorporeal membrane oxygenation and SCD

Hou et al. conducted a retrospective cohort study on patients with acute fulminant myocarditis who had received extracorporeal membrane oxygenation (ECMO) treatment due to a poor response of conventional regimens. In brief, the authors suggest that an early initiation of ECMO can potentially improve outcomes and reduce complications in this very specific patient group. This is especially interesting since recent data did not paint a favorable picture for ECMO in cardiogenic shock originated from myocardial infarction (7), and also in refractory cardiac arrest (8). Before the background of such data discouraging the routine use of ECMO, the question arises if we shouldn't rather concentrate our efforts and resources on other aspects beside elaborating elaborate ECMO schemes. Probably, investing into specialized patient subgroups (e.g., fulminant myocarditis) on the one-, and other improvements around resuscitation (e.g., mandatory basic life support education in schools or AED programs) on the other hand could be more useful. Apart from resource allocation, the potentially severe side effects of ECMO which can massively impact on patients' daily lives if they survive (9, 10), must not be forgotten. In this Research Topic, indeed, Wu et al. present a case report of ECMO treatment for myocardial infarction with cardiac arrest and CPR, who developed gangrenous cholecystitis as a complication.

## Neurological survival

Neurological function is one of the main determinants of a successful CPR (11); however, respective assessments can be challenging, and still little is known in various cardiac arrest subgroups. Fuchs et al. retrospectively assessed data of intraoperative cardiac arrest, and found that it is a rare event, but on the other hand more likely in older individuals and such with a high American Society of Anesthesiologists (ASA classification) status, in cardiac and vascular surgery, and in emergency procedures. In most cases, ROSC can be achieved, and 30-days survival as well as favorable neurological outcome are likely if CPR is conducted immediately—This highlights the necessity to act quickly, and provides a goal that we could potentially also achieve in OHCA if the respective systems would be strengthened adequately.

## SCD after hospital discharge

Hopefully, acute coronary syndromes (potentially leading to initial SCD) are treated successfully, and patients are discharged home again. However, certain sequelae still play a role in the “aftermath”, for instance myocardial scar tissue or heart failure, in turn maybe leading to a proarrhythmic state (12, 13). Choi et al. conducted a prospective registry study for this patient group. They found that Killip class ≥3, chronic kidney disease stage ≥4, severe anemia, cardiopulmonary support, no dual antiplatelet therapy at discharge, and left ventricular ejection fraction ≤ 35% were independent predictors for early cardiac death within 90 days after the initial acute coronary syndrome event. In the context of the present body of literature (14), these prognosticators could in the future be validated and developed further to finally inform clinicians about a population at high risk for SCD who might profit from early defibrillator implantation.

To conclude, cardiac arrest research must be further promoted to successfully impact on affected patients—the overall goal is to find improved treatment solutions, boost survival rates, and to develop strategies for optimal post-cardiac arrest care and rehabilitation of survivors. Of note, resource allocation will be key to address the right issues at the right time.

## Author contributions

SS: Conceptualization, Supervision, Writing – original draft, Writing – review & editing. EB: Writing – review & editing. PS: Supervision, Writing – review & editing.
